# Ningxiang pig-derived *lactobacillus reuteri* modulates host intramuscular fat deposition via branched-chain amino acid metabolism

**DOI:** 10.1186/s40168-024-02013-6

**Published:** 2025-01-31

**Authors:** Mei Yang, Qian Xie, Jing Wang, Andong Zha, Jiashun Chen, Qian Jiang, Meng Kang, Qiuchun Deng, Yulong Yin, Bie Tan

**Affiliations:** 1 Hunan Provincial Key Laboratory for the Products Quality Regulation of Livestock and Poultry, College of Animal Science and Technology, Hunan Agricultural University, Changsha, Hunan 410128 P. R. China; 2 Yuelushan Laboratory, Changsha, Hunan 410128 P. R. China; 3 Key Laboratory of Agro-Ecological Processes in Subtropical Region, National Engineering Laboratory for Pollution Control and Waste Utilization in Livestock and Poultry Production, Institute of Subtropical Agriculture, Chinese Academy of Sciences, Changsha, Hunan 410125 P. R. China

## Abstract

**Background:**

Gut microbiota has been extensively demonstrated to modulate host lipid metabolism. Higher intramuscular fat (IMF) accumulation in Chinese indigenous breed pigs is associated with their special gut microbiota structure. However, the specific microbes and metabolic pathways responsible for lipid deposition are still poorly understood.

**Results:**

In the present study, a comparative analysis of the gut microbiota and metabolome in obese Ningxiang (NX) pigs and lean Duroc × Landrace × Yorkshire (DLY) pigs was conducted. The results revealed a higher abundance of gut lactobacilli and a correlation of branched-chain amino acid (BCAA) metabolism pathway in NX pigs. We proceeded to verify the roles of various lactobacilli strains originating from NX pigs in BCAA metabolism and lipids deposition in SD rats. We demonstrated that *L. reuteri* is a fundamental species responsible for modulating lipid deposition in NX pigs and that increased circulating levels of BCAA are positively linked to greater lipid deposition. Additionally, it has been verified that *L. reuteri* originating from NX pigs has the ability to improve BCAA synthesis in the gut and enhance IMF content in lean DLY pigs. The expression of genes related to lipid synthesis was also significantly upregulated.

**Conclusions:**

Taken together, our results imply that NX pig-derived *L. reuteri* regulates BCAA metabolism and plays a potential role in improving the meat quality of lean pig breeds through modulation of host intramuscular lipid deposition. The results provide a new strategy for improving the meat quality of commercial pigs by influencing host metabolism through supplementing dietary additives.

Video Abstract

**Supplementary Information:**

The online version contains supplementary material available at 10.1186/s40168-024-02013-6.

## Introduction

The current focus of swine production has transitioned from prioritizing quantity and production efficiency to improving product quality due to changing consumer demands [1]. There are a range of factors that impact meat quality, of which intramuscular fat (IMF) content is a crucial indicator and is strongly linked to pork tenderness and flavor [2]. Furthermore, diet and breed have been identified as primary determinants in the formation of meat quality traits [3]. Chinese indigenous pig breeds are well-known for their exceptional meat quality, which is distinguished by elevated IMF values and desirable meat color [4].

The gut microbiota undergoes mutual selection and adaptation with the host [5]. Previous study has shown that Chinese indigenous pig breeds, such as the obese Sanjiang pigs exhibit distinctive metabolic and microbial profiles compared to lean Yorkshire pigs, which could be linked to their diverse metabolic phenotypes [6]. Growing evidence suggests a causal relationship between gut microbiota and host metabolic phenotypes, potentially mediated through metabolites derived from the gut microbiota [7]. Certain gut microbiota-derived metabolites have exhibited either anti-obesity or pro-obesity effects [8]. For instance, short-chain fatty acids have been found to mitigate lipid accumulation [9]. Conversely, trimethylamine-N-oxide has been demonstrated to expedite fat deposition [10]. Gut microbiota is also a potential regulatory factor in amino acid homeostasis, and the correlations of BCAA profiles and gut microbiota have to be identified in some disease models [11].

Branched-chain amino acids (BCAAs) have well been demonstrated to regulate lipid metabolism and increase IMF content in pigs [12, 13]. BCAA metabolism may serve as a potential mechanistic link of gut microbiota influencing muscle fat accumulation [11]. Therefore, the present study was conducted to analyze the correlation between gut microbial composition and BCAA metabolism using obese Ningxiang (NX) pigs and commercially available lean Duroc × Landrace × Yorkshire (DLY) pigs. To further establish the causal connection between lactobacilli and branched-chain amino acid metabolism, *L. reuteri* was isolated from NX pigs crucial bacterial biomarker that elevated circulating and ileal contents levels of BCAAs. Additionally, the substantial presence of *L. reuteri* from NX pigs facilitated the promotion of IMF deposition in DLY pigs by influencing BCAA metabolism.

## Materials and methods

### Experimental design

The experimental procedures received approval from the Institutional Animal Care and Use Committee of Hunan Agricultural University.

A total of sixty finishing pigs including thirty DLY pigs and thirty NX pigs were used in this study. DLY pigs with similar genetic backgrounds and body conditions were individually housed in the finishing barn of the farm of Hunan Longhua Agricultural and Livestock Development Co Ltd., Chaling County, Zhuzhou City, Hunan Province. NX pigs with similar genetic backgrounds were reared at the farm of Hunan Chuweixiang Agricultural and Animal Husbandry Co., Ltd.. NX and DLY pigs were fed according to their respective feeding standards, respectively. All pigs were free to drink water, and the feeding management was carried out uniformly according to the company’s breeding norms. Ileal mucosa, colonic contents, and muscle were isolated and collected for analysis.

Fecal samples were obtained from NX finishing pigs and used to isolate *L. mucosae*, *L. reuteri*, and *L. salivarius*. The samples were suspended in phosphate-buffered saline (PBS) buffer and then serially diluted to 10^−8^. Different diluted samples were plated on MRS agar and were subjected to a temperature of 37 ℃ for 2 days in an anaerobic workstation, with a gas mixture comprising 80% N_2_, 10% CO_2_, and 10% H_2_. A solitary colony was picked from the plates and refined via streaking of the bacterial colony on modified MRS agar. The single strain’s 16S rRNA gene was amplified with two universal primers (27F: 5′-AGAGTTTGATCCTGGCTCAG-3′ and 1492R: 5′-TACGGCTACCTTGTTACGACTT-3′), followed by sequencing through the Sanger method. The 16S rRNA gene sequences were then aligned with the NCBI nucleotide sequence database to distinguish three strains. Isolated strains′ growth curves were determined. Isolated strains were utilized in the experiments conducted on rat and pig cultures, as shown in Fig. S1. Furthermore, the *L. reuteri* XY227 isolated from NX pig fecal samples was reserved at the China Center for Type Culture Collection (CCTCC M 20231546 XY227). *L. reuteri* XY227 was subsequently processed into powder form by CRVAB Bio-technology Co., Ltd.

Male SD rats, 7 weeks old, were procured from Boresen Biotech Company (Hunan, China). The rats were accommodated in Individual Ventilated Cages (IVCs) that maintained a controlled temperature of 23–25 ℃ with a 12-h light–dark cycle. They had unrestricted access to food and water. After acclimatizing for one week, the rats were assigned randomly to four groups of ten replicates each and one rat per replicate. NC group comprised of rats administered oral sterile saline (1 mL), LM group comprised of rats administered oral *L. mucosae* (1 mL, 10^9^ CFU/mL); LR group comprised of rats administered oral *L. reuteri* (1 mL, 10^9^ CFU/mL); LS group comprised of rats administered oral *L. salivarius* (1 mL, 10^9^ CFU/mL). All groups were treated once daily for 8 weeks. The body weight (BW) of the rat was measured by weighing the rat on a scale for one week. Blood was sampled from the heart and serum was collected to measure BCAA levels. The inguinal white adipose tissue (iWAT), epididymal white adipose tissue (eWAT), perirenal adipose tissue (PAT), brown adipose tissue (BAT), gastrocnemius muscle, ileal contents, and feces were quickly frozen and stored at − 80 °C.

Sixteen castrated DLY pigs with an average initial body weight of 63.36 ± 1.28 kg were randomly divided into two groups, with each group consisting of eight replicates and one pig per replicate. One group was fed a normal diet (NC), while the other group was given LR, which contained 0.4% (w/w) *L. reuteri* (1 × 10^11^ CFU/kg normal diet). The diets were formulated to meet the nutrient requirements recommended by the NRC (2012). Pellet feed was used in the experiment and bacteria powder and feed were mixed evenly before feeding. The pigs were kept in a typical environment with a natural light regime, 50% to 60% humidity, and a temperature of 25 ± 5 ℃. No antibiotics were given in advance to the pigs to comply with the antibiotic-free production standard. Throughout the entirety of the experiment, the pigs had free access to food and water. The treatments were consistently administered for a duration of 50 days. Blood samples were centrifuged at 3500 × *g* for 10 min at 4 °C, and the supernatants were stored at − 80 °C for subsequent analysis. Samples of longissimus dorsi muscle (LDM), jejunum, ileum, ileal contents, and colonic contents were collected and immediately frozen in liquid nitrogen. They were then stored at − 80 °C for future analysis.

### DNA isolation and 16S rRNA amplicon sequencing

Microbial genomic DNA was extracted from the ileal mucosa and colonic contents of NX and DLY pigs via CTAB according to the manufacturer’s protocol. The DNA was quantified using NanoDrop (Thermo Fisher Scientific, Wilmington, DE, USA), and the universal primers 341F (5′-CCTACGGGNGGCWGCAG-3′) and 805R (5′-GACTACHVGGGTATCTAATCC-3′) were utilized to amplify the V3–V4 hypervariable regions of the 16S rRNA genes. The PCR amplification was conducted using the following optimal conditions: initial denaturation at 98 °C for 30 s, followed by 32 cycles of denaturation at 98 °C for 10 s, annealing at 54 ℃ for 30 s, and extension at 72 ℃ for 45 s. A final extension at 72 ℃ for 10 min was performed. The size of the PCR products was confirmed by performing 2% agarose gel electrophoresis.

The amplicon pools underwent sequencing preparation, with the size and quantity of the amplicon library assessed utilizing an Agilent 2100 Bioanalyzer (Agilent, USA) and the Library Quantification Kit for Illumina (Kapa Biosciences, Woburn, MA, USA), respectively. The NovaSeq PE250 platform was employed to sequence the libraries, whilst the LC-Bio provided manufacturer recommendations for sequencing the samples on an Illumina NovaSeq platform. Paired-end reads were assigned to samples based on their unique barcode and subsequently truncated by removing the barcode and primer sequence. The paired-end reads were then merged using FLASH software. Quality filtering on the raw reads was performed with specific filtering conditions to obtain high-quality clean tags according to fqtrim (version 0.94). Chimeric sequences were filtered out using Vsearch software (version 2.3.4). Finally, after dereplication using DADA2, a feature table and feature sequence were obtained. Alpha and beta diversity were calculated by random normalization of the same sequences. The feature abundance was normalized using the relative abundance of each sample, according to the SILVA (release 138) classifier. Alpha diversity, which analyses the species diversity complexity of a sample through the calculation of five different indices (Shannon, Simpson, Chao1, Pielou-e), was determined using QIIME2. The beta diversity was also calculated by QIIME2, and the R package was used to produce graphs. Sequence alignment was performed using Blast, and annotations of feature sequences for each representative sequence were obtained from the SILVA database. Diagrams were produced using the R package (v3.5.2). Linear discriminant analysis (LDA) effect size (LEfSe) analysis was used to identify the differentially abundant genera between the two groups. Microbial functions were predicted using PICRUSt2 against KEGG databases using default parameters.

### Metabolomic analysis

The muscle and colonic contents samples (30 ~ 100 mg) were homogenized with an equal volume of PBS and centrifuged at 12,000 × *g* for 5 min. The supernatant was transferred to a clean tube and mixed with an equal volume of methanol. The mixture was centrifuged again at 12,000 × *g* for 10 min and the supernatant was carefully transferred to a new tube. The sample is then transferred to a vacuum concentrator (SPD130P1, Thermo Fisher, USA) for complete drying. A solution of 200 µL of ethyl acetate: water (1:1) is added to fully dissolve the concentrated supernatant. The sample is then centrifuged at 12,000 × *g* for 5 min at 4 °C, and the supernatant is carefully transferred to a sample vial for ultra-high-resolution liquid chromatography-mass spectrometry (LC–MS) analysis. The samples were analyzed using the analytical system (Q-Exactive Plus, Thermo Fisher, USA). Metabolic pathways and networks were identified through database searches (KEGG, http://www.genome.jp/kegg/) and pathway analysis. The resulting metabolic differences between the two groups were mapped to their respective biochemical pathways. Metabolic pathways can be classified according to the biochemical reactions or functions they perform. Metabolic pathway enrichment analysis typically involves the identification of a set of metabolites that participate in a specific biochemical process.

### Biochemical analysis

Serum concentrations of triglyceride (TG), total cholesterol (TC), high-density lipoprotein (HDL), and low-density lipoprotein (LDL) were analyzed using an automatic biochemical instrument (KHB 450, Shanghai Kehua bio-engineering Co., Ltd.).

### RNA extraction and quantification

Total RNA was extracted from gastrocnemius, longissimus dorsi muscle, jejunum, and ileum tissues using RNAiso Plus (Cat # 9109, Takara Biomedical Technology (Beijing) Co., Ltd.). Subsequently, cDNA was synthesized with Evo M-MLV RT Kit with gDNA Clean for qPCR (AG11705, Accurate Biotechnology (Hunan) Co., Ltd., Changsha, China) in accordance with the manufacturer’s instructions. Real-time PCR was conducted using SYBR Green Premix Pro Taq HS Qpcr Kit (AG11701, Accurate Biotechnology (Hunan) Co., Ltd., Changsha, China) with a real-time PCR instrument (LightCycler480II, Roche, Germany). The ΔCt method was applied to analyze the results, which were further normalized to reference genes.

### Quantification of bacterial load by q-PCR

Genomic DNA from feces was extracted using a Seno fecal genomic DNA extraction kit (China), following the manufacturer’s instructions. The quantities of *L. reuteri* bacterial species present in the fecal matter were analyzed via quantitative real-time PCR, as previously reported [14]. A standard curve was created by measuring Cq values for dilutions of the template DNA concentration, ranging from 10^9^ to 10^1^ copies/μL. Based on this curve, the copy numbers of the target bacteria were calculated.

### Determination of IMF and amino acid

Intramuscular fat (IMF) content was assessed using the Soxtec Extraction method with petroleum ether. Freeze-dried digesta samples (100 mg) were homogenized with 5 mL of 0.02 mol/L HCl using ultrasonic extraction for 30 min. After 15 min of centrifugation at a speed of 14,000 × *g*, the supernatant was decanted, and the process was repeated two to three times. The collected supernatants were mixed to achieve a constant volume. One milliliter of the filtrate and serum were mixed thoroughly with an equal volume of 8% sulfosalicylic acid and left to rest overnight. Afterward, they were centrifuged at a temperature of 4 ℃ for 10 min at a rate of 10,000 × *g*. The resulting supernatant was then filtered through a membrane with a pore size of 0.22 μm. Finally, the filtrate was utilized foramino acid analysis by using an Automatic Amino Acid Analyzer (HITACHI, Japan, LA8080).

### Oil red staining

Frozen sections of the LDM were made using a Frozen Microtome (Leica, Germany). Frozen sections were removed from the − 20℃ refrigerator and returned to room temperature. The frozen sections were stained using oil red staining solution for 8–10 min. After that, they were differentiated with 60% isopropanol, washed with pure water, and counterstained with hematoxylin for 3–5 min. Finally, they were differentiated with a differentiation solution for 2–8 s.

### Branched-chain α-ketoacids (BCKA) quantification

Cold methanol (80 μL) was added to 20 μL serum. The mixture was vortexed and then centrifuged at 14,000 × *g* for 15 min at 4 °C. The supernatant was collected and evaporated to dryness in a vacuum centrifugal concentrator. For the tissue samples, 50–100 mg of frozen tissue and 500 μL of cold methanol were homogenized and sonicated in an ice water bath. It was followed by centrifugation at 14,000 × *g* for 15 min at 4 °C. The supernatant was collected, concentrated, and dried. The remaining tissue precipitate was resuspended in 500 μL of cold methanol, and the above steps were repeated. The stated process was applied to obtain the serum and tissue-dried samples. The dried samples were treated with 500 μL of O-phenylene diamine (2-Keto-3-methylbutyric acid-^13^C_5_ sodium salt), and the mixture was sonicated for 15 min, vortexed, and then stirred at 80 °C for 30 min. The mixture was allowed to cool, and 800 μL of ethyl acetate was added to the vortex for 5 min. The mixture was then centrifuged at 14,000 × *g* for 10 min at 4 °C, and 500 μL of the supernatant was concentrated and dried. The initial tubes were extracted again with 800 μL of ethyl acetate, then vortexed and centrifuged at 14,000 × *g* for 10 min at 4 ℃. The supernatant (500 μL) was then aspirated and saved from each sample. Subsequently, the supernatant was completely dried and derivatized using a 0.1M 50:50 blend of ammonium acetate and methanol, with ultrasound for 10 min followed by vortexing until the precipitate was suspended. The samples were ultimately spun at 14,000 × *g* for 15 min at 4 ℃. Following this, 100 μL of the supernatant was transferred to brown sample vials to undergo LC–MS analysis.

### Statistical analysis

The results are presented as the mean and SEM and visualized using the GraphPad Prism 8.0 software (GraphPad Software, San Diego, Canada). Statistical significance was determined by using a two-tailed Student’s *t*-test or one-way ANOVA test. The Kruskal–Wallis test was implemented for non-normally distributed data. Any dissimilarities were considered statistically significant at *P* < 0.05.

## Results

### *Lactobacillus* was the main differential microbiota between DLY and NX pigs and correlated with BCAA metabolism

Our research group found that NX pigs have a noticeably higher intramuscular and body fat content compared to imported commercial DLY pigs [15]. To determine whether the superior fat deposition performance of NX pigs is related to microbial function and metabolic function, we first investigated the differences in microbial and metabolic functions of colonic contents between obese NX pigs and lean DLY pigs and found that NX pigs harbor a distant gut microbiota compared with lean DLY pigs (Fig. S1A). Furthermore, the microbial function of the KEGG pathways was then predicted by LEfSe (Fig. S1B). The result revealed that secondary bile acid biosynthesis, BCAA biosynthesis, and aromatic amino acids biosynthesis were enriched in NX pigs. We next used nontargeted metabolomic analysis to investigate the primary differential metabolic pathways of muscle and colonic contents between NX pigs and DLY pigs were the BCAA synthesis and degradation pathways (Fig. S1C–D).

To further explore the key microbiota differences between the two breeds, we compared the microbiota of the ileal mucosa and colonic contents of NX and DLY pigs. There was no notable variation in the mucosal and luminal α-diversity, except for the colonic luminal Simpon index and Pielou-e which displayed a remarkable increase (Fig. S2A–B). A principal coordinates analysis (PCoA) based on Bray–Curtis was used to investigate the overall structure of the ileal mucosal and colonic luminal microbiota and showed a separation in the gut microbiota structure among NX pigs with DLY pigs (Fig. S2C–D). Additionally, a comparative analysis of microbial composition at the genus level between NX and DLY pigs was conducted using hierarchical clustering and heatmap analyses. The heatmap and hierarchical cluster tree showed a distinct distinction in the ileal mucosal and colonic luminal microbiota communities between DLY pigs and NX pigs (Fig. S2E–F), as confirmed by the separated distance within the group. LEfSe analysis was utilized to identify bacterial biomarkers that discriminate between NX and DLY pigs at the genus level, with an LDA score threshold of 3.0. In the mucosa, the percentage of the genera *Lactobacillus*, *HT002*, *Ligilactobacillus*, and *Limosilactobacillus* was significantly higher in the NX pigs (Fig. S3A). LEfSe analysis also demonstrated that the NX pigs were characterized by the bacterial genera *UCG_005*, *Lactobacillus*, *Clostridium_sensu_stricto_1*, and *Agathobacter* in the colonic contents (Fig. S3B). *Lactobacillus* was determined to be the distinguished genus between NX and DLY pigs. Additionally, the abundance of *Lactobacillus* showed a strong positive correlation with serum BCAA concentrations (Fig. S3C–D). Based on the above data, pathways associated with BCAA metabolism were found to be enriched in NX pigs, potentially originating from the gut microbiota with *Lactobacillus*.

### NX pig-derived *L. reuteri*, but not *L. salivarius* or *L. mucosae*, elevates the fat accumulation in rats

**Fig. 1:**
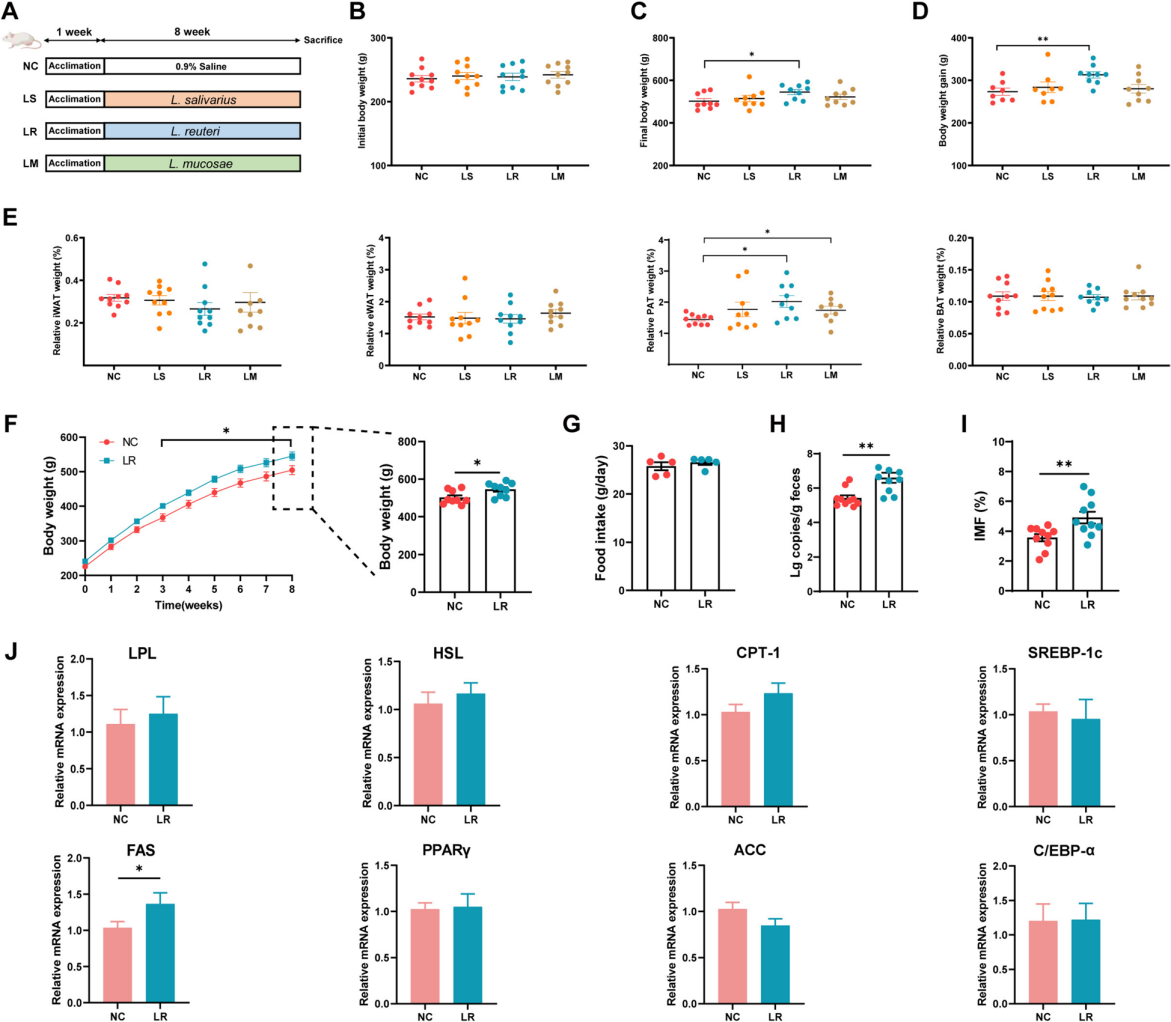
*L. reuteri* promotes fat accumulation in rats. **A** Schematic of the experiment for key bacterial rescue. **B** Initial body weight. **C** Final body weight. **D** Body weight gain. **E** Relative iWAT, eWAT, pWAT, BAT weight. **F** Body weight. **G** Copy number of *L. reuteri* in the rat’s feces. **H** Food intake. **I** Intramuscular fat content of gastrocnemius muscle. **J** The mRNA levels of muscle lipid metabolism-related genes.*P<0.05, **P <0.01,^ **#**^*P*<0.001

To elucidate the role of *Lactobacillus* in lipid metabolism, we isolated and cultured *L. salivarius*, *L. reuteri*, and *L. mucosae* in vitro from fecal samples of finishing NX pigs (Fig. S4). Subsequently, we investigated the potential causal relationship between *Lactobacillus* isolated from the experimental pigs, BCAA metabolism, and the accumulation of IMF in the host by conducting a gavage experiment using *L. salivarius*, *L. reuteri*, and *L. mucosae* in rats (Fig. 1A). To ascertain the impact of *Lactobacillus* on SD rats, the study examined the BW and weight of iWAT, eWAT, and pWAT. It was observed that there were no significant differences in initial BW among the four groups during the gavage phase (Fig. 1B). However, the final BW and BW gain were significantly increased in rats treated with *L. reuteri*, but not *L. salivarius* or *L. mucosae*, in comparison to control treatment (Fig. 1C–D). There was a marked increase in the relative weight of pWAT when treated with *L. reuteri* and *L. mucosae* in comparison to rats treated with the vehicle (Fig. 1E). The final weight of rats supplemented with *L. reuteri* was significantly higher than that of the control group (Fig. 1F), and there was no significant difference in feed intake between the two groups (Fig. 1G). The weight gain of rats in the LR group could be ruled out as caused by the increase in food intake. These findings indicated a significant boost in the absolute abundance of *L. reuteri* in the feces of LR rats (Fig. 1H), demonstrating successful colonization of *L. reuteri* in the rat intestinal tracts. Furthermore, our data demonstrated that *L. reuteri* supplementation greatly increased the IMF content of the gastrocnemius muscle in rats (Fig. 1I). The expression of multiple genes linked to lipid metabolism in the gastrocnemius muscle was analyzed, with a particular focus on *HSL*, *CPT-1*, *SREBP-1C*, *FAS*, *PPARγ*, and *ACC*. The mRNA expression of *FAS* was subsequently found to be significantly up-regulated by the administration of *L. reuteri* (Fig. 1J). No differences, however, were noted in the expression of the remaining genes between the NC and LR rats. Overall, these data provided evidence of *L. reuteri* regulating lipid metabolism in LR rats.

### NX pig-derived *L. reuteri* reprogrammed the intestinal BCAA metabolism and absorption

**Fig. 2:**
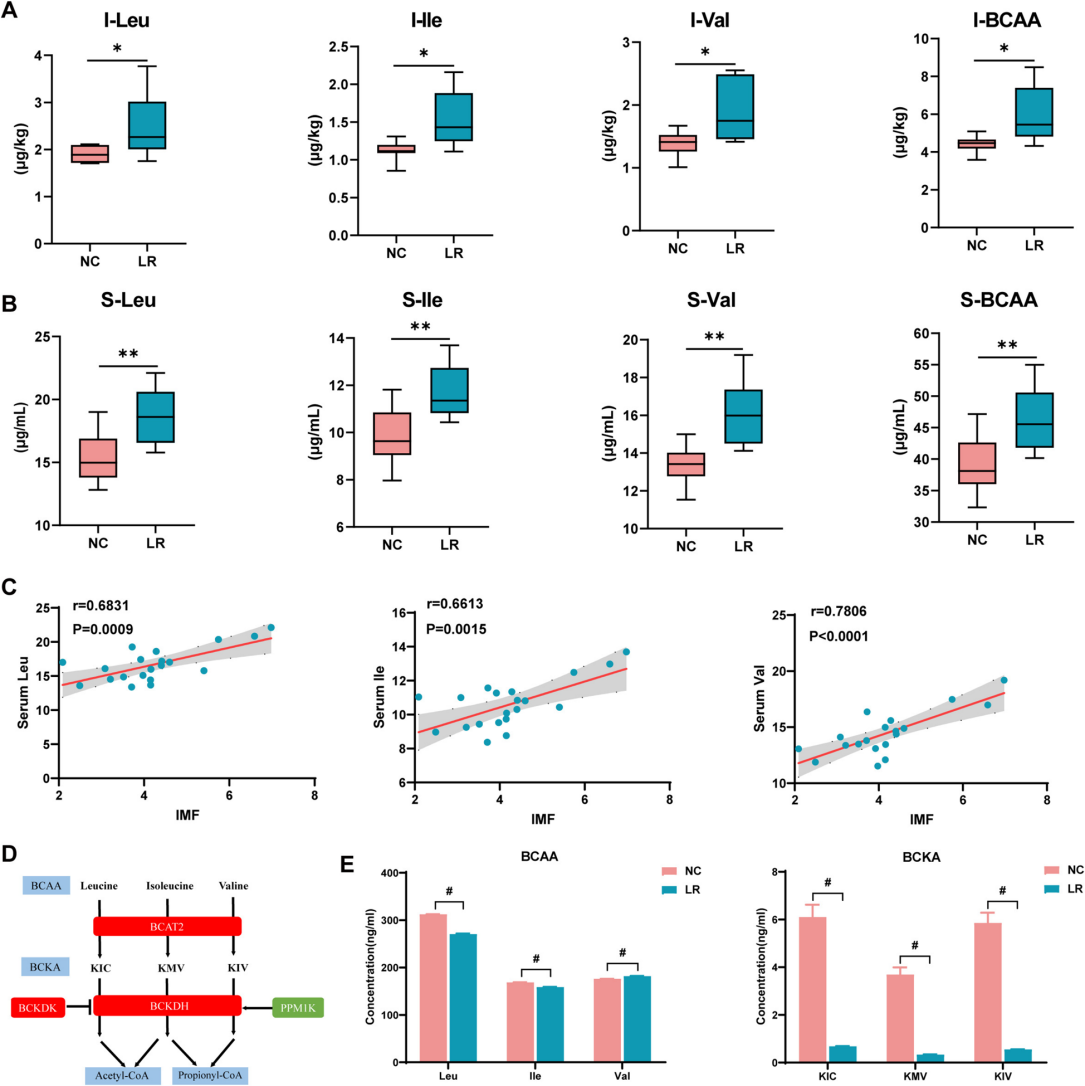
*L. reuteri* reprogrammed the intestinal BCAA metabolism. The concentrations of BCAA in the **A** ileal contents and **B** serum of rats. **C** Serum BCAA concentration and its association with IMF within gastrocnemius muscle of rats. **D** BCAA metabolic pathway. **E** BCAA and BCKA concentrations of *L. reuteri* in the MRS medium.*P<0.05, **P<0.01, ^**#**^*P*<0.001

Gut microbiota metabolites and components function as signaling molecules to regulate the lipid-related endocrine system or directly impact metabolic organs [16]. Given the differential abundance of *L. reuteri* between NX and DLY pigs and the enriched pathways associated with BCAA biosynthesis in NX pigs, we investigated the potential influence of *L. reuteri* on intestinal BCAA metabolism. Rats treated with *L. reuteri* exhibited elevated ileal BCAA levels compared to the NC group (Fig. 2A). Subsequently, an investigation focused on the gut microbiota’s contribution to serum BCAA levels, with LR rats showing elevated serum BCAA levels compared with normal controls (Fig. 2B). Furthermore, a positive correlation was found between serum BCAA concentration and IMF content in rats (Fig. 2C), suggesting a critical role of BCAA in fat accumulation.

BCAA can be converted into BCKAs (Fig. 2D). To confirm the capability of *L. reuteri* to metabolize BCAA, we performed an in vitro fermentation of *L. reuteri* in an MRS medium and measured the levels of BCKA and BCAA after 24 h. The concentration of BCAA was altered, accompanied by changes in BCKA after incubation, which supports the ability of *L. reuteri* to influence BCAA metabolism (Fig. 2E). We then explored the effects of LR on BCAA metabolism and fat deposition using a finishing pig model.

**Fig. 3:**
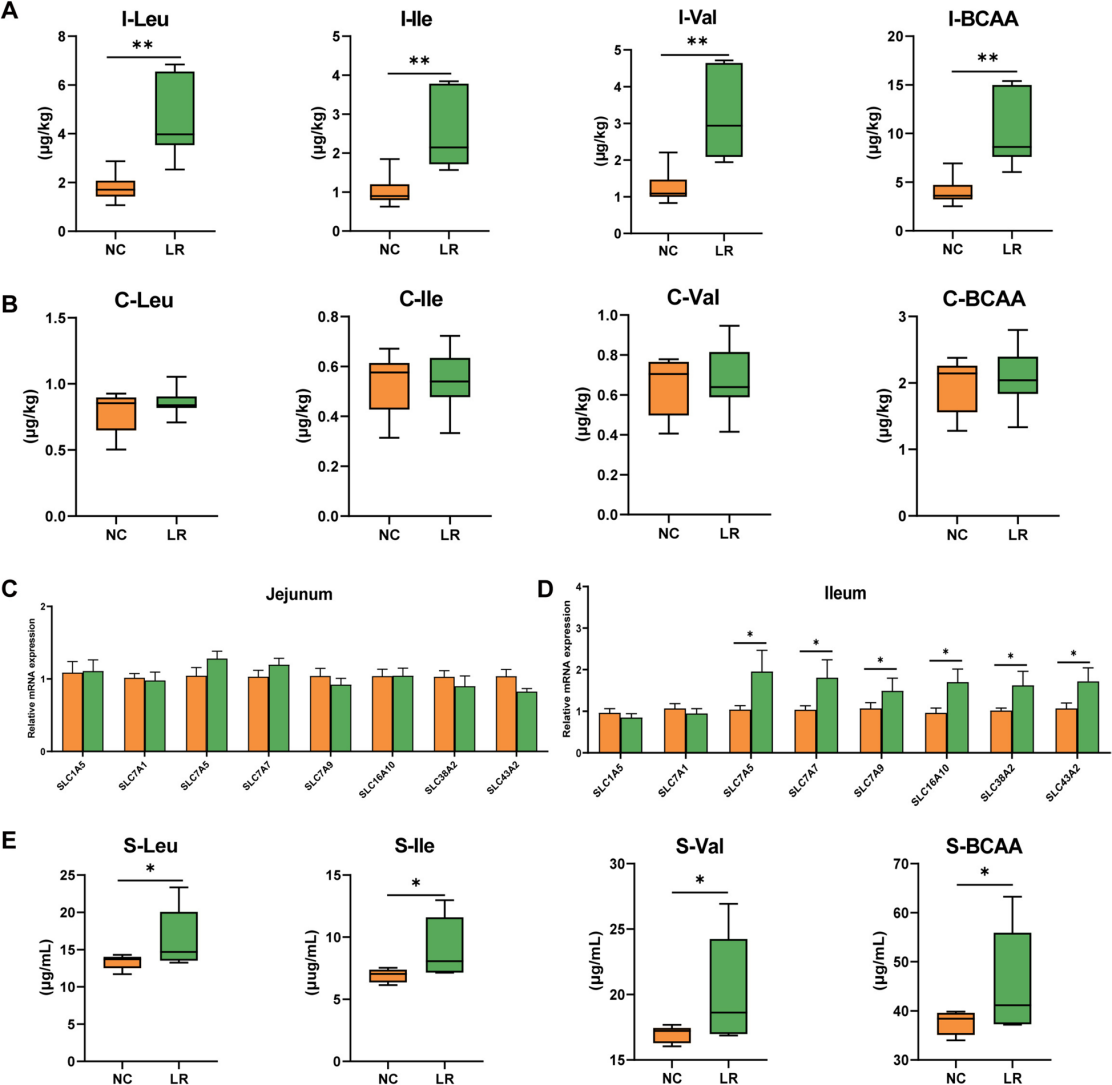
*L. reuteri* promotes the synthesis and absorption of intestinal BCAA in DLY pigs. BCAA levels in the **A** ileal and **B** colonic contents. The mRNA levels of amino acid transporter protein in the **C** jejunum and **F** ileum. **E** Serum BCAA levels.*P<0.05, **P<0.01, ^**#**^*P*<0.001

Consistent with rats, pigs supplemented with LR showed enhanced BCAA levels in the ileum (Fig. 3A). Metabolomics analysis revealed increased BCKA levels in the ileal contents of *L. reuteri*-treated pigs, indicating the influence of *L. reuteri* on the metabolism of BCAA in the gut (Fig. S5C). Colonic content analysis showed no significant difference in BCAA and BCKA levels between *L. reuteri*-treated and vehicle-treated groups (Fig. 3B; Fig. S5D). The primary origin of amino acids in the body is dietary, which is absorbed and digested in the intestine [17]. As a result, we analyzed the genes related to intestinal amino acid transporters and found that no significant difference was observed in the jejunum, but higher expression in the ileum of LR pigs (Fig. 3C–D). Key BCAA catabolic genes exhibited no difference in the jejunum but higher *BCAT2* expression in the ileum of *L. reuteri*-supplemented pigs (Fig. S5A–B).

#### Intestinal BCAA metabolic alternations by *L. reuteri* influenced circulating BCAA and then regulated muscle IMF deposition

**Fig. 4:**
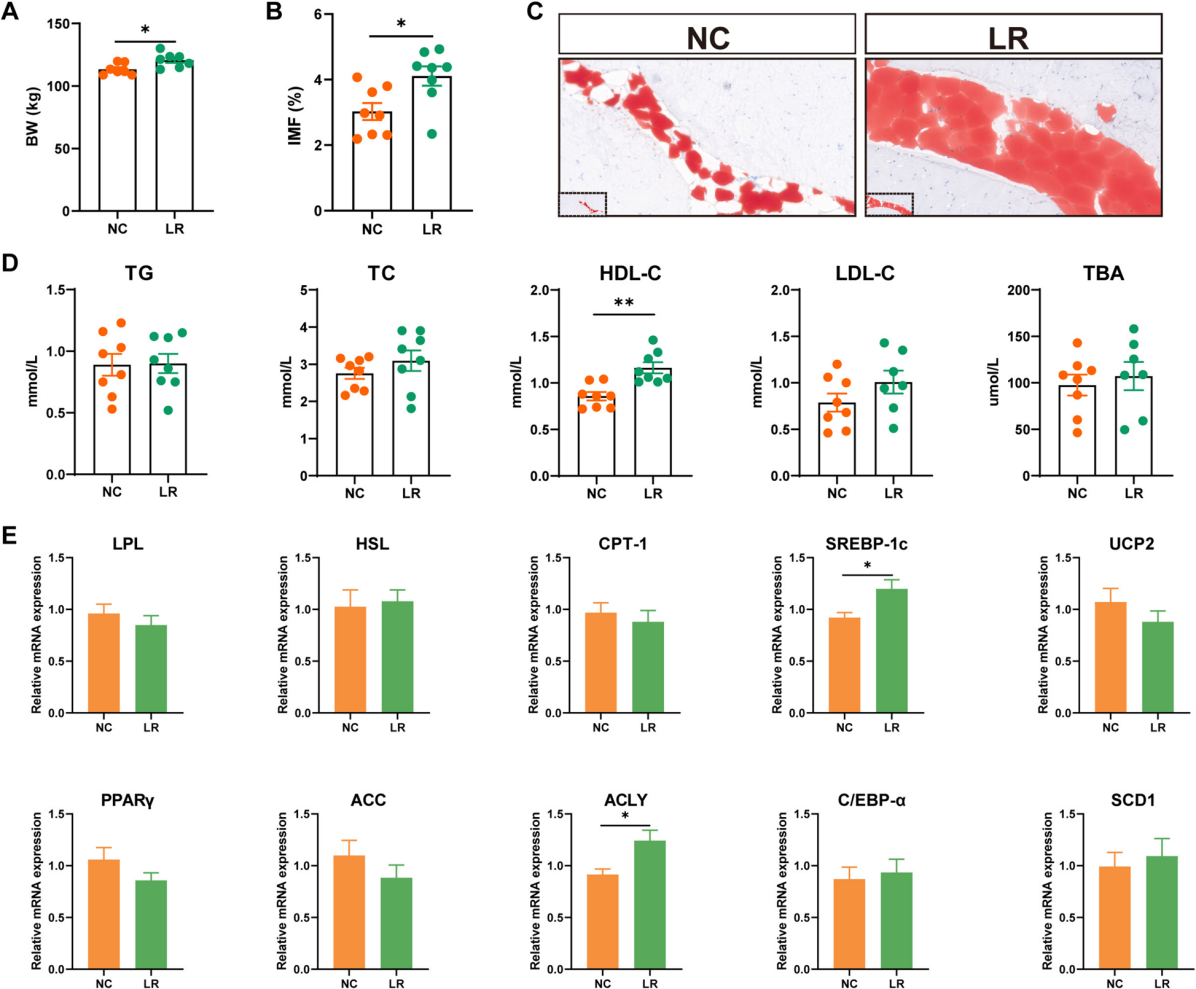
*L. reuteri* elevated muscle IMF contents of DLY pigs. **A** Body weight. **B** The content of IMF in the LDM. **C** Oil red staining of the LDM. **D** Serum lipid levels. **E** The mRNA levels of muscle lipid metabolism-related genes.*P<0.05, **P <0.01, ^**#**^*P*<0.001

A strong association between elevated circulating BCAA and obesity has been repeatedly observed in human and rodent models, we analyzed the concentrations of the circulation BCAA [18]. The results demonstrated that serum BCAA levels were expectedly higher in LR pigs (Fig. 3E). To reveal the impact of individual *L. reuteri* on growth, lipid deposition, and BCAA metabolism, we conducted NX pig-derived *L. reuteri* on DLY finishing pigs for 50 days, while final BW and IMF in muscle were markedly enhanced in *L. reuteri*-fed DLY pigs (Fig. 4A–C), indicating a role in the improvement in muscle meat quality and enhancement of lipid metabolism. We further quantified lipid metabolism-related genes in the muscle, there was *SREBP-1C* and *ACLY* showed a higher expression level, which was consistent with the increased IMF content in the *L. reuteri* supplemented pigs (Fig. 4E). As shown in Fig. 4D, L*. reuteri* significantly increased serum HDL-C levels, but had little effect on the serum levels of TG, TC, LDL-C, and TBA compared with the control diet group. Our present findings supported the improvement potential of *L. reuteri* in raising IMF in muscle and showed that *L. reuteri*-regulated intestinal BCAA metabolism serves as the underlying mechanism for the lipid accumulation efficacy of *L. reuteri*.

## Discussion

The available evidence strongly suggests a pivotal role of gut microbiota in modulating lipid metabolism [19, 20]. Distinct fat deposition patterns are evident between obese indigenous pigs and lean commercial pigs [15, 21]. Comparing Chinese Indigenous (obese) and commercial (lean) pigs reveals entirely different microbial communities, with Indigenous pigs exhibiting attributes such as superior meat quality and high IMF content [3, 4]. In this study, a notable enrichment of *Lactobacillus* species, specifically *L. salivarius*, *L. reuteri*, and *L. mucosae*, was observed in NX pigs. *L. salivarius* and *L. mucosae* have demonstrated anti-obesity and anti-atherosclerotic effects in animal experiments [22, 23]. Additionally, *L. reuteri* was found to metabolize tryptophan into indole-3-aldehyde, enhancing the body’s anti-tumor immunity [24].

To our knowledge, the impact of *L. reuteri* on BCAA metabolism has not been reported. Emerging evidence supports the crucial role of gut microbiota in BCAA metabolism [25, 26]. Our research validated that there is enrichment in the BCAA metabolic pathway in NX pig breeds. Research has indicated that obese-related bacteria have been shown to have higher BCAA synthesis rates and lower BCAA breakdown rates [27, 28]. Fecal microbiota transplantation from obese individuals into germ-free mice induced obesity with a significant increase in circulating BCAA levels [29]. Gastric administration of *L. reuteri* isolated from NX pigs increased rat final body weight and pWAT weights, accompanied by elevated ileal and circulating BCAA levels. Thus, the results suggest that *L. reuteri* promotes body lipid deposition and reshapes gut BCAA metabolism. Further research is essential to investigate their interrelation.

In other studies, it has been observed that elevated serum BCAA levels in obese individuals are associated with gut microbiota possessing enriched BCAA synthesis capability [26]. Moreover, specific gut bacteria, particularly the BCAA-producing phenotype (referred to as *Prevotella copri*), demonstrated an overall increase in serum BCAA, inducing a higher degree of obesity in high-fat diet mice [11]. Previous in vitro experiments have confirmed that gut microbiota can degrade BCAA into branched-chain fatty acids , exerting a protective effect against atherosclerosis[30]. Further in vitro experiments have demonstrated that the addition of *L. reuteri* to the MRS culture medium influences the BCAA and BCKA content, leading us to infer that the elevated levels of both intestinal and circulating BCAA result from *L. reuteri* promotion of BCAA metabolism. Our research provides evidence supporting the causative relationship between gut microbiota-mediated BCAA metabolism and lipid deposition, presenting a promising strategy for improving lipid deposition in commercial pigs.

In good accordance with our results, previous studies have also shown that the overexpression of branched-chain ketoacid dehydrogenase kinase in the liver during BCAA metabolism can increase ATP-citrate lyase phosphorylation and stimulate de novo fat synthesis. The gut microbiota fundamentally influences skeletal muscle development and lipid metabolism characteristics [31]. The gut microbiota essentially influences skeletal muscle development and lipid metabolism characteristics [32–34]. This influence on skeletal muscle development and lipid metabolism is further underscored in our study where DLY pigs fed with LR exhibited significantly increased circulating BCAA levels compared to the control group. The functional role of LR in BCAA synthesis increases gut BCAA levels, accompanied by elevated expression of amino acid transporter-related genes. Simultaneously, increased IMF content and highly expressed fat synthesis genes were observed in LR-fed pigs, supporting LR-mediated BCAA metabolism as a potential mechanism influencing lipid metabolism. Our evidence for LR promoting IMF deposition in NX pig breeds supports the notion that gut microbiota-mediated BCAA synthesis metabolism is causally related to lipid deposition. Essential amino acids, such as BCAA, cannot be synthesized by the host and must be obtained from the diet or produced by gut microbiota [35]. Moreover, a high-BCAA diet leads to reduced body weight gain by decreasing food intake, suggesting that supplementing probiotics with BCAA synthesis functionality is another method for improving meat quality [36]. BCAA metabolism is closely associated with obesity, with increased levels of circulating BCAA and their metabolites positively correlating with obesity [18, 37]. BCKA, the metabolite of BCAA, was increased with it in this work. Little is known about the physiological functions of BCKA compared to BCAA. In one study, BCKA was shown to re-establish HFD-induced obesity in mitochondrial branched-chain aminotransferase knockout mice [38].

These findings highlight the role of BCAA and their metabolites as key signaling factors that influence host metabolism and gut microbiota. Further studies are required to explore the mechanisms linking BCAAs to muscle lipid deposition. Additionally, ongoing research may reveal new applications of *Lactobacillus roidii* in enhancing BCAA utilization and improving meat quality. However, it is important to note that this study has limitations, particularly in terms of sample size. Further research with larger cohorts and more advanced sequencing technologies will be essential to validate and extend these findings.

## Conclusion

In summary, this study provides strong evidence that NX pigs and DLY pigs have distinct microbiota compositions and BCAA metabolism capabilities. Furthermore, *L. reuteri* may represent one of the key species in the microbiota that influences IMF deposition and BCAA metabolism in pigs. Our research offers evidence supporting the causal relationship between gut microbiota, BCAA metabolism, and lipid deposition, providing valuable insights for improving meat quality in commercial pigs.

## Supplementary Information

Supplementary Material 1: Figure S1.The difference of gut microbial function and metabolic function between DLY and NX pigs and correlated with BCAA metabolism. (A) Bray–Curtis PCoA plots of the colonic contents microbiota composition. (B) Differences in gut microbial functions (LDA>2.5) predicted by KEGG and analyzed by LEfSe. Differential metabolite enrichment pathways in the (C) muscle and (D) colonic contents. Figure S2. Alpha diversity estimates of microbiota community by Shannon, Simpon, Chao1, and Pielou-e index of ileal mucosa (A) and colonic contents (B). Bray–Curtis PCoA plots of (C) the ileal mucosa and (D) colonic contents microbiota composition. (E) Heatmap of cluster analysis of genus in the ileal mucosa microbial community between NX and DLY pigs. (F) Clustering analysis of the colonic microbial composition at genus level, the hierarchical cluster tree on the left represents the clustering of subjects, the bar plot on the right represents the relative abundance of the bacterial genus.*P<0.05, **P<0.01, ^**#**^*P*<0.001 Figure S3. *Lactobacillus* was the main differential microbiota between DLY and NX pigs and correlated with BCAA metabolism. LEfSe representing taxa at the genus level (LDA score ≥ 3) enriched in (A) ileal mucosa and (B) colonic contents. Serum BCAA (Leu, Ile, Val) concentration and its association with relative abundance of *Lactobacillus* within the ileal mucosa (C) and colonic contents (D) of NX and DLY pigs. Figure S4. Growth curve (mean OD600nm) of (A) *L.reuteri*, (B)*L.salivarius* and (C)*L. mucosae* in MRS medium. Figure S5. Gene expression of BCAA metabolic enzymes in (A) jejunum and (B) ileum. The concentrations of BCKA in (C) ileal and (D) colonic contents.*P<0.05, **>*P*<0.01, ^#^*P*<0.001

## Data Availability

The sequences generated in this study are available in the NCBI Sequence Read Archive database (Accession Number: PRJNA1066614, PRJNA1066501).
