# Supplementary figures and images for: Ningxiang pig-derived lactobacillus reuteri modulates host intramuscular fat deposition via branched-chain amino acid metabolism
Source: Microbiome. 2025 Jan 31;13:32. doi: 10.1186/s40168-024-02013-6 (PMC11786426; doi:10.1186/s40168-024-02013-6)

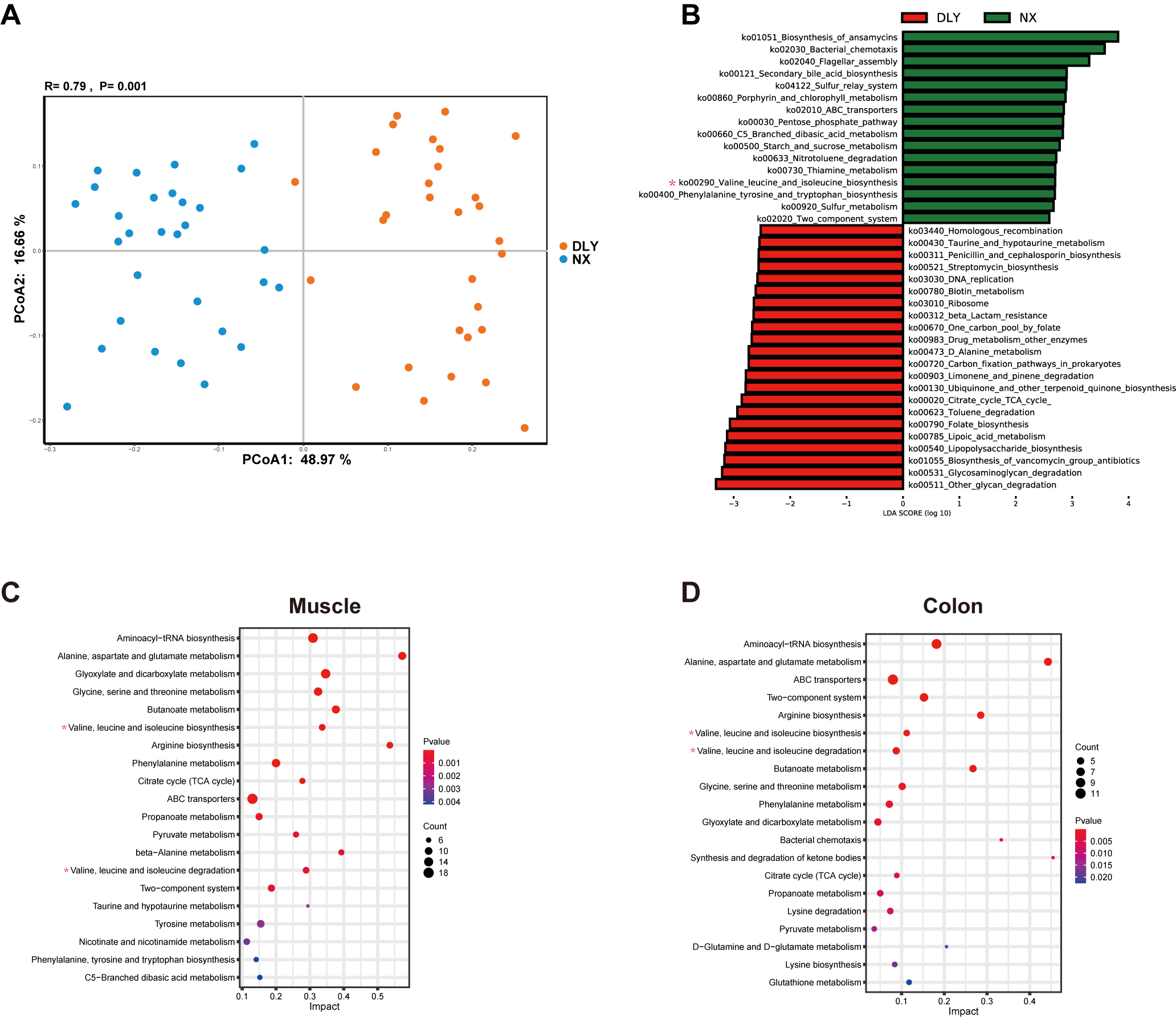

Supplement: Supplementary file 2 — Supplementary Material 1: Figure S1.The difference of gut microbial function and metabolic function between DLY and NX pigs and correlated with BCAA metabolism. (A) Bray–Curtis PCoA plots of the colonic contents microbiota composition. (B) Differences in gut microbial functions (LDA>2.5) predicted by KEGG and analyzed by LEfSe. Differential metabolite enrichment pathways in the (C) muscle and (D) colonic contents. Figure S2. Alpha diversity estimates of microbiota community by Shannon, Simpon, Chao1, and Pielou-e index of ileal mucosa (A) and colonic contents (B). Bray–Curtis PCoA plots of (C) the ileal mucosa and (D) colonic contents microbiota composition. (E) Heatmap of cluster analysis of genus in the ileal mucosa microbial community between NX and DLY pigs. (F) Clustering analysis of the colonic microbial composition at genus level, the hierarchical cluster tree on the left represents the clustering of subjects, the bar plot on the right represents the relative abundance of the bacterial genus.*P<0.05, **P<0.01, #P<0.001 Figure S3. Lactobacillus was the main differential microbiota between DLY and NX pigs and correlated with BCAA metabolism. LEfSe representing taxa at the genus level (LDA score ≥ 3) enriched in (A) ileal mucosa and (B) colonic contents. Serum BCAA (Leu, Ile, Val) concentration and its association with relative abundance of Lactobacillus within the ileal mucosa (C) and colonic contents (D) of NX and DLY pigs. Figure S4. Growth curve (mean OD600nm) of (A) L.reuteri, (B)L.salivarius and (C)L. mucosae in MRS medium. Figure S5. Gene expression of BCAA metabolic enzymes in (A) jejunum and (B) ileum. The concentrations of BCKA in (C) ileal and (D) colonic contents.*P<0.05, **>P<0.01, #P<0.001 [file 40168_2024_2013_MOESM1_ESM.zip › Fig.S1.tif]

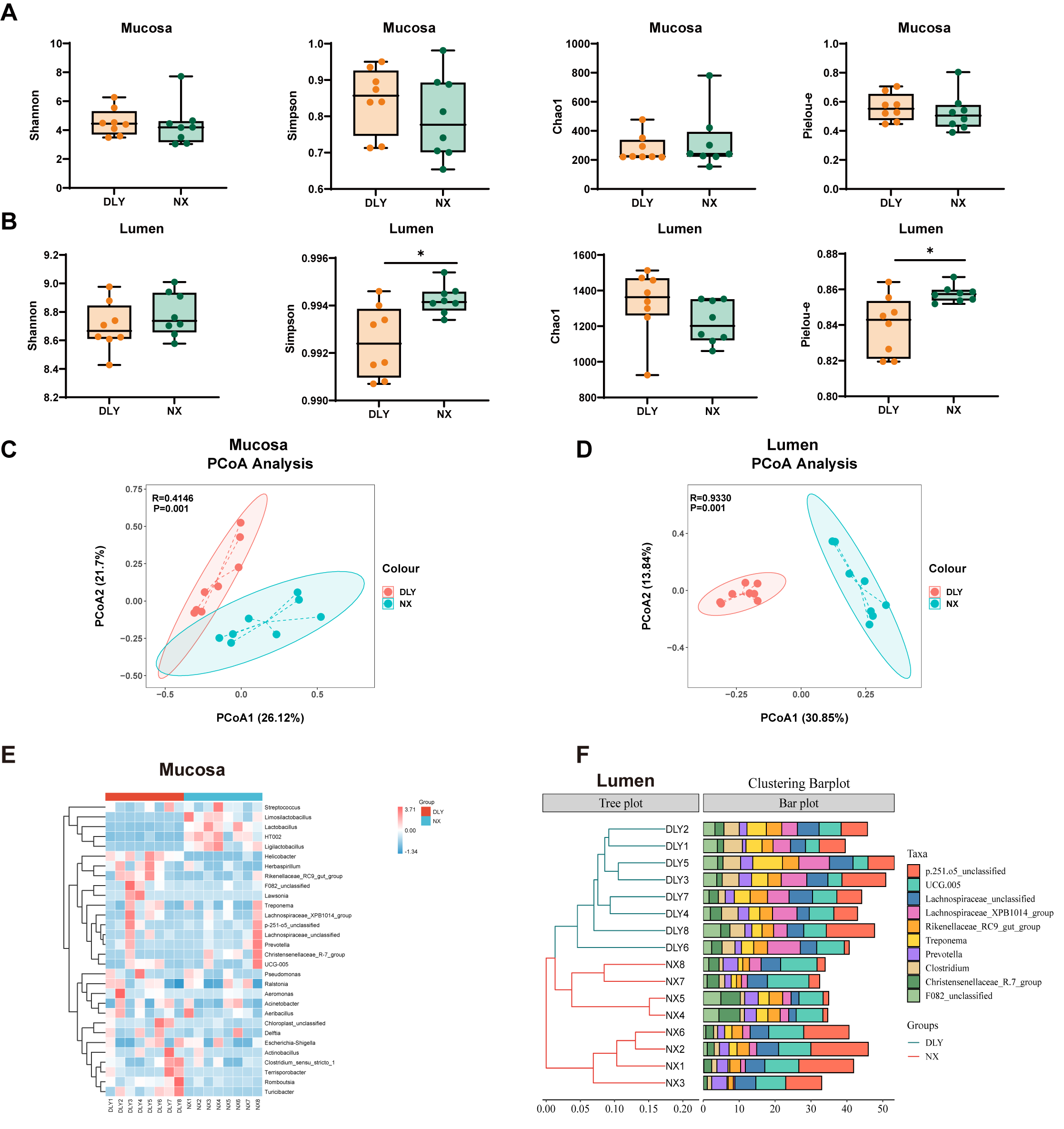

Supplement: Supplementary file 2 — Supplementary Material 1: Figure S1.The difference of gut microbial function and metabolic function between DLY and NX pigs and correlated with BCAA metabolism. (A) Bray–Curtis PCoA plots of the colonic contents microbiota composition. (B) Differences in gut microbial functions (LDA>2.5) predicted by KEGG and analyzed by LEfSe. Differential metabolite enrichment pathways in the (C) muscle and (D) colonic contents. Figure S2. Alpha diversity estimates of microbiota community by Shannon, Simpon, Chao1, and Pielou-e index of ileal mucosa (A) and colonic contents (B). Bray–Curtis PCoA plots of (C) the ileal mucosa and (D) colonic contents microbiota composition. (E) Heatmap of cluster analysis of genus in the ileal mucosa microbial community between NX and DLY pigs. (F) Clustering analysis of the colonic microbial composition at genus level, the hierarchical cluster tree on the left represents the clustering of subjects, the bar plot on the right represents the relative abundance of the bacterial genus.*P<0.05, **P<0.01, #P<0.001 Figure S3. Lactobacillus was the main differential microbiota between DLY and NX pigs and correlated with BCAA metabolism. LEfSe representing taxa at the genus level (LDA score ≥ 3) enriched in (A) ileal mucosa and (B) colonic contents. Serum BCAA (Leu, Ile, Val) concentration and its association with relative abundance of Lactobacillus within the ileal mucosa (C) and colonic contents (D) of NX and DLY pigs. Figure S4. Growth curve (mean OD600nm) of (A) L.reuteri, (B)L.salivarius and (C)L. mucosae in MRS medium. Figure S5. Gene expression of BCAA metabolic enzymes in (A) jejunum and (B) ileum. The concentrations of BCKA in (C) ileal and (D) colonic contents.*P<0.05, **>P<0.01, #P<0.001 [file 40168_2024_2013_MOESM1_ESM.zip › Fig.S2.tif]

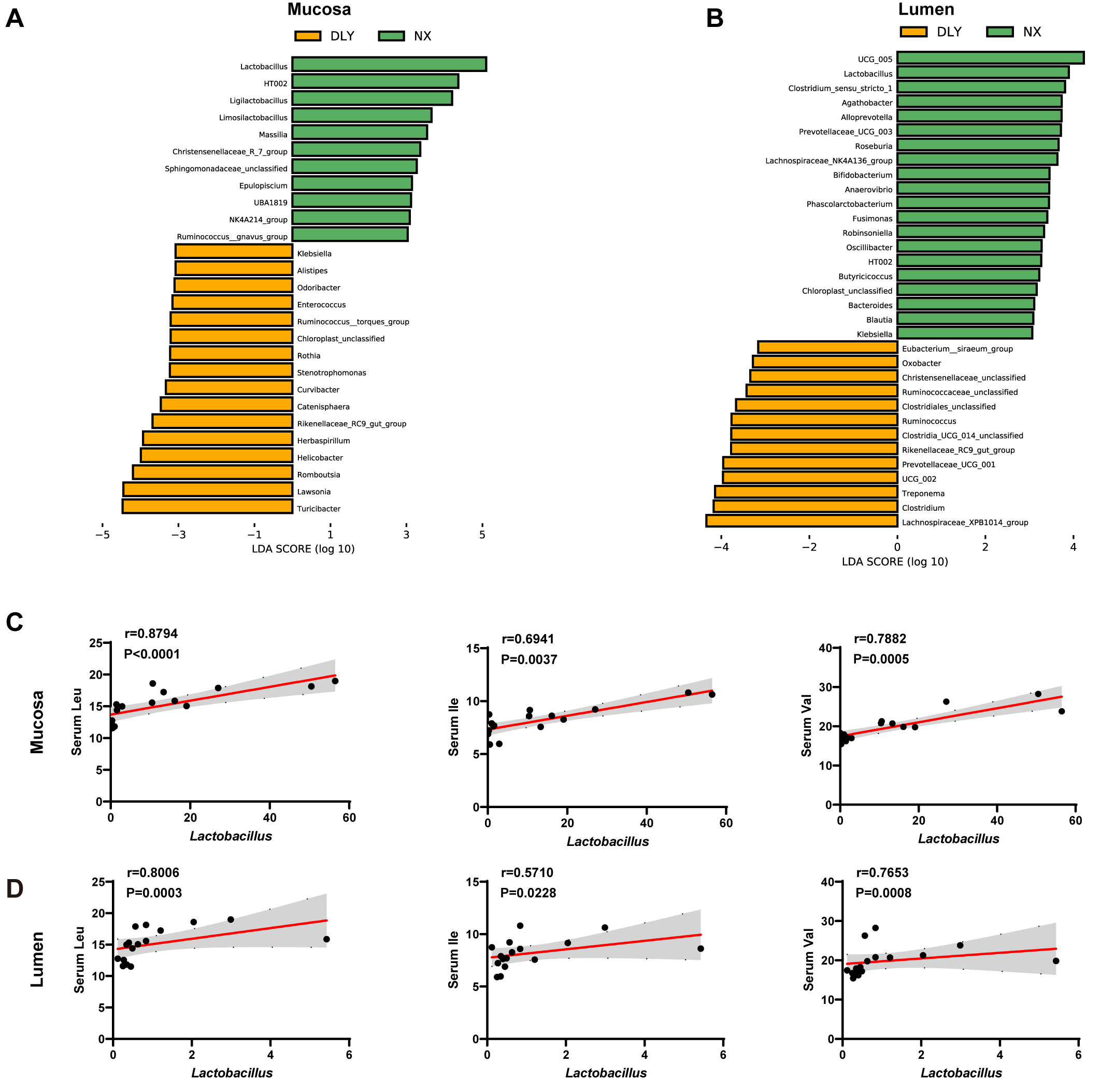

Supplement: Supplementary file 2 — Supplementary Material 1: Figure S1.The difference of gut microbial function and metabolic function between DLY and NX pigs and correlated with BCAA metabolism. (A) Bray–Curtis PCoA plots of the colonic contents microbiota composition. (B) Differences in gut microbial functions (LDA>2.5) predicted by KEGG and analyzed by LEfSe. Differential metabolite enrichment pathways in the (C) muscle and (D) colonic contents. Figure S2. Alpha diversity estimates of microbiota community by Shannon, Simpon, Chao1, and Pielou-e index of ileal mucosa (A) and colonic contents (B). Bray–Curtis PCoA plots of (C) the ileal mucosa and (D) colonic contents microbiota composition. (E) Heatmap of cluster analysis of genus in the ileal mucosa microbial community between NX and DLY pigs. (F) Clustering analysis of the colonic microbial composition at genus level, the hierarchical cluster tree on the left represents the clustering of subjects, the bar plot on the right represents the relative abundance of the bacterial genus.*P<0.05, **P<0.01, #P<0.001 Figure S3. Lactobacillus was the main differential microbiota between DLY and NX pigs and correlated with BCAA metabolism. LEfSe representing taxa at the genus level (LDA score ≥ 3) enriched in (A) ileal mucosa and (B) colonic contents. Serum BCAA (Leu, Ile, Val) concentration and its association with relative abundance of Lactobacillus within the ileal mucosa (C) and colonic contents (D) of NX and DLY pigs. Figure S4. Growth curve (mean OD600nm) of (A) L.reuteri, (B)L.salivarius and (C)L. mucosae in MRS medium. Figure S5. Gene expression of BCAA metabolic enzymes in (A) jejunum and (B) ileum. The concentrations of BCKA in (C) ileal and (D) colonic contents.*P<0.05, **>P<0.01, #P<0.001 [file 40168_2024_2013_MOESM1_ESM.zip › Fig.S3.tif]

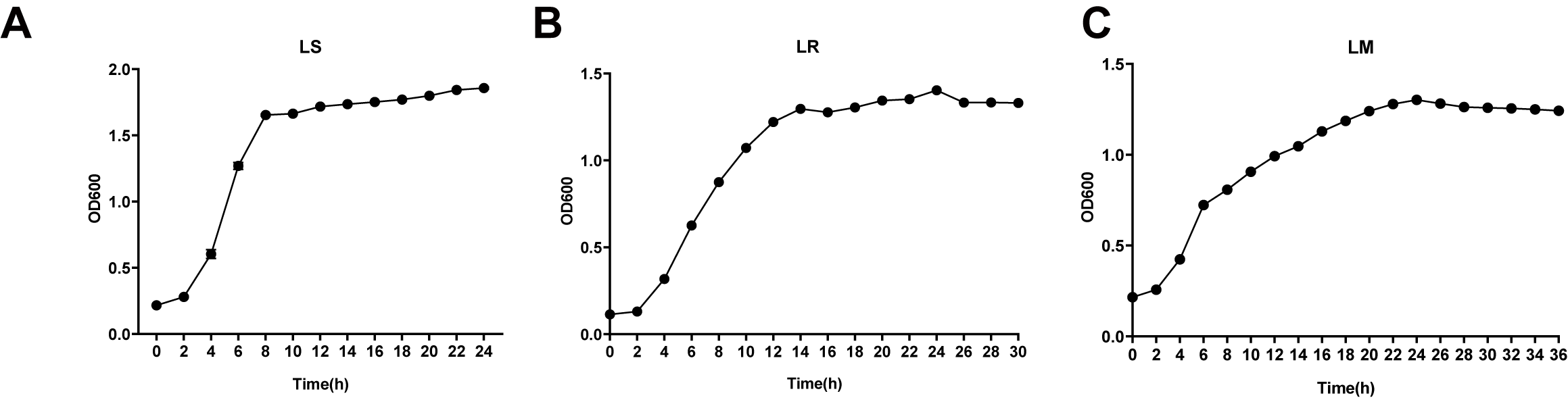

Supplement: Supplementary file 2 — Supplementary Material 1: Figure S1.The difference of gut microbial function and metabolic function between DLY and NX pigs and correlated with BCAA metabolism. (A) Bray–Curtis PCoA plots of the colonic contents microbiota composition. (B) Differences in gut microbial functions (LDA>2.5) predicted by KEGG and analyzed by LEfSe. Differential metabolite enrichment pathways in the (C) muscle and (D) colonic contents. Figure S2. Alpha diversity estimates of microbiota community by Shannon, Simpon, Chao1, and Pielou-e index of ileal mucosa (A) and colonic contents (B). Bray–Curtis PCoA plots of (C) the ileal mucosa and (D) colonic contents microbiota composition. (E) Heatmap of cluster analysis of genus in the ileal mucosa microbial community between NX and DLY pigs. (F) Clustering analysis of the colonic microbial composition at genus level, the hierarchical cluster tree on the left represents the clustering of subjects, the bar plot on the right represents the relative abundance of the bacterial genus.*P<0.05, **P<0.01, #P<0.001 Figure S3. Lactobacillus was the main differential microbiota between DLY and NX pigs and correlated with BCAA metabolism. LEfSe representing taxa at the genus level (LDA score ≥ 3) enriched in (A) ileal mucosa and (B) colonic contents. Serum BCAA (Leu, Ile, Val) concentration and its association with relative abundance of Lactobacillus within the ileal mucosa (C) and colonic contents (D) of NX and DLY pigs. Figure S4. Growth curve (mean OD600nm) of (A) L.reuteri, (B)L.salivarius and (C)L. mucosae in MRS medium. Figure S5. Gene expression of BCAA metabolic enzymes in (A) jejunum and (B) ileum. The concentrations of BCKA in (C) ileal and (D) colonic contents.*P<0.05, **>P<0.01, #P<0.001 [file 40168_2024_2013_MOESM1_ESM.zip › Fig.S4.tif]

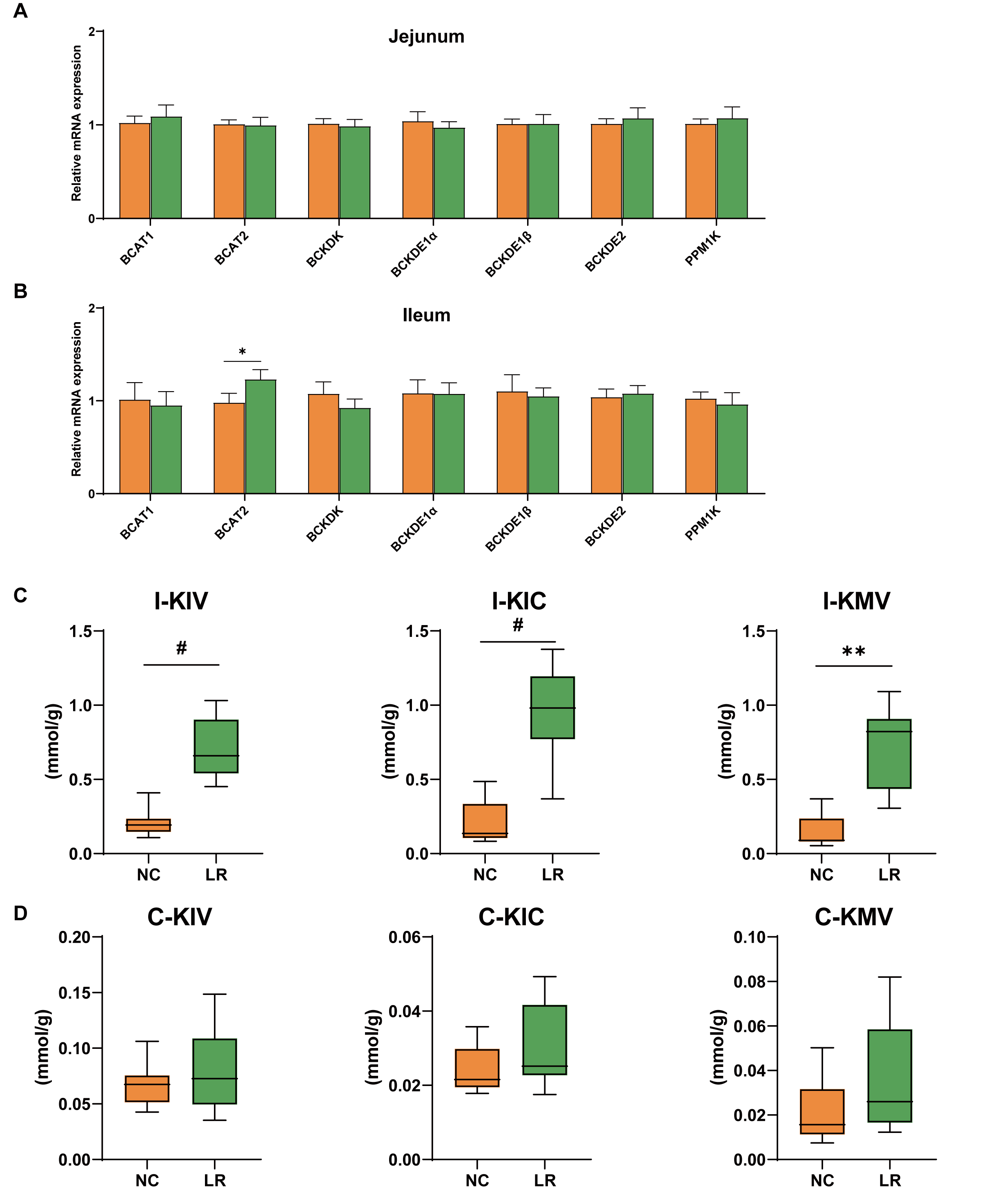

Supplement: Supplementary file 2 — Supplementary Material 1: Figure S1.The difference of gut microbial function and metabolic function between DLY and NX pigs and correlated with BCAA metabolism. (A) Bray–Curtis PCoA plots of the colonic contents microbiota composition. (B) Differences in gut microbial functions (LDA>2.5) predicted by KEGG and analyzed by LEfSe. Differential metabolite enrichment pathways in the (C) muscle and (D) colonic contents. Figure S2. Alpha diversity estimates of microbiota community by Shannon, Simpon, Chao1, and Pielou-e index of ileal mucosa (A) and colonic contents (B). Bray–Curtis PCoA plots of (C) the ileal mucosa and (D) colonic contents microbiota composition. (E) Heatmap of cluster analysis of genus in the ileal mucosa microbial community between NX and DLY pigs. (F) Clustering analysis of the colonic microbial composition at genus level, the hierarchical cluster tree on the left represents the clustering of subjects, the bar plot on the right represents the relative abundance of the bacterial genus.*P<0.05, **P<0.01, #P<0.001 Figure S3. Lactobacillus was the main differential microbiota between DLY and NX pigs and correlated with BCAA metabolism. LEfSe representing taxa at the genus level (LDA score ≥ 3) enriched in (A) ileal mucosa and (B) colonic contents. Serum BCAA (Leu, Ile, Val) concentration and its association with relative abundance of Lactobacillus within the ileal mucosa (C) and colonic contents (D) of NX and DLY pigs. Figure S4. Growth curve (mean OD600nm) of (A) L.reuteri, (B)L.salivarius and (C)L. mucosae in MRS medium. Figure S5. Gene expression of BCAA metabolic enzymes in (A) jejunum and (B) ileum. The concentrations of BCKA in (C) ileal and (D) colonic contents.*P<0.05, **>P<0.01, #P<0.001 [file 40168_2024_2013_MOESM1_ESM.zip › Fig.S5.tif]
